# Towards Starting a Hand Transplant Unit and Achieving Success in a Hand Transplant: The Standard Operating Procedure

**DOI:** 10.1055/s-0043-1776435

**Published:** 2024-02-29

**Authors:** Vinita Puri, Narasiman Venkateshwaran, Raghav Shrotriya, Chandrashekhar Chalwade

**Affiliations:** 1 Department of Plastic Surgery, Seth GS Medical College and KEM Hospital, Mumbai, Maharashtra, India

**Keywords:** forearm, hand, hand transplant, vascularized composite allotransplantation, microsurgery, amputation, surgical, standard operating protocol

## Abstract

Vascularized Composite Tissue Allotransplantation (VCA) allows replacement of lost body parts from brain-dead donors. These surgeries are laborious, time-intensive, and require vast planning. With the advent of better immunosuppressants, VCA will increasingly play an important role in the reconstructive field. In this paper, the authors share their standard operating protocol created after much deliberation.

Hand is an extremely important part of a human being's body. Second only to the brain, the human hand may be responsible for a lot of the advancement that humankind has made over the course of evolution. The importance of the human hand can be gauged from the fact that the loss of a hand accounts for up to 60% loss of function for the whole person. 1

Patients may suffer the loss of upper limb due to avulsion injuries, industrial accidents, electrical burns, traffic accidents, or congenital causes. Those with congenital absence of whole or a part of the upper limb may develop suitable adaptations and are functionally better adapted than those who lose the limb suddenly in an accident. This is all the more challenging in patients who have suffered bilateral amputations. With microsurgery techniques in their armamentarium, plastic surgeons are in a unique position to help these patients. Replantation of amputated body parts has been performed by plastic surgeons routinely since more than 50 years now. Vascularized Composite Tissue Allotransplantation (VCA) allows replacement of lost limbs from brain-dead donors. These surgeries are laborious, time-intensive, and require vast planning for ensuring the optimum use of the available resources and a successful outcome. The authors performed India's 11th hand transplant on August 11, 2021. This paper describes the efforts taken and the standard operating procedure (SOP) created by the department for this extensive endeavor. It should be noted that the exact procedure may vary as per the local regulations in each country. Hand transplant is more technically known as hand and upper limb reconstruction using vascularized composite allotransplantation. 2 For the purpose of this paper, the term “hand transplant” will refer to upper limb transplants at various levels—arm, forearm, wrist, or partial hand.

## Philosophy of Hand Transplant

A hand transplant is *not* a lifesaving procedure like a renal or liver transplant but is definitely a procedure which improves the patient's life significantly in terms of psychosocial health, daily functionality, earning prospects, and mental well-being. Since allotransplants mandate the use of lifelong immunosuppressants accompanied by their ill effects, any transplant procedure needs to be titrated in its benefits with the potential risks associated with prolonged use of immunosuppression.

The hands have such a complex structure and hold a position of importance that their replenishment in the form of transplantation has been deemed to be worth the immunosuppression-related risks, since the expected functional gain with a transplant is significantly more than a prosthesis (especially below elbow). All transplant patients should be chosen after extensive counselling sessions so that they understand the risk–benefit ratio.

## When to Embark upon it: Prerequisites for Having a Transplant Unit

The journey on the path of starting a transplant program starts with the conviction and the desire of a dedicated team coupled with a robust institutional support since it requires inputs from both. It is a truly multidisciplinary endeavor. Each country will have their own rules and regulations and it is important to know them before embarking on the path to transplant. A transplant team will include a lead team of plastic surgeons along with orthopaedic surgeons, anesthesiologists, nephrologist, pathologist, operation theater ancillary staff, nurses, therapists, blood bank, and social worker. It is helpful if the hospital already has a functional solid organ transplant program. 3 Once the decision is taken to start the VCA, the first step is applying for a license which is provided by the appropriate state health authority. The process of applying requires extensive paperwork and is basically meant to ensure that minimum standards are maintained in terms of personnel and infrastructure. Inspection for the transplant license is also an important step and preparedness for the same cannot be emphasized enough. Once the license is procured, the preparation commences.

### Preparation

Though performing a transplant is technically quite similar to performing a hand replant which most plastic and reconstructive surgeons will be well versed with, there are a lot of different issues which require careful planning and thought. All members of the team need to be up-to-date with the latest in the field including immunosuppression, management of a rejection episode, etc. It includes reading up the peer-researched literature, attending workshops and conferences, being a part of peer groups of fellow transplant surgeons (local/national associations) and gaining information from other centers, online sources, even seeking guidance from national and international leaders in the field (e.g., Iyer et al and Sharma et al, have described their journey of setting up India's first hand transplant unit in a four-part article series). 4 5 Attending cadaveric workshops for harvest and practice of transplant surgery will also add to the training and create additional harvest teams which will be beneficial to all. Presently, the authors regularly conduct these cadaveric courses.

It is important to publicize the availability of this service in your hospital. Again, this needs to be done taking into account the prevalent rules and regulations in every country. Public awareness campaigns, information on the institutional website and social media handles may help in attracting potential patients to your Outpatient Department (OPD). It will be helpful if there is a particular time or day of OPD dedicated to these patients. Once a patient consults regarding hand transplant, he/she needs to be explained the full-scale procedure, requirement of lifelong immunosuppression, the costs involved, and the need to stay near the hospital from the time of registration to almost 1 year after the transplant. In our experience, this is a lot of information to absorb in the first consultation and up to two to three counselling sessions are required before the patients and the family members can take an informed decision of going ahead with this procedure.

A patient willing to go ahead will be investigated, referred to many different specialists (most importantly nephrologists), and start arranging for funds. Crowdfunding and help from nongovernmental organizations should be sought at this point for helping the patient with the finances. Depending on the prevalent health care structure and insurance system, the need for the funding should be individualized to the local context. It is helpful to have a checklist of the various things to be completed before the patient is registered ( Supplementary Materials S1 and S2 , available in the online version). Registration of the patient with the state appropriate transplant authority is a watershed event. It means the patient gets into the transplant waiting list and the patient and surgeons have to be ready to go ahead with the procedure anytime that the donor may become available after that. Therefore, all the basic preparation should have been completed before registering and thereafter maintenance preparations like three monthly blood investigations of the patient, three monthly team discussion and patient-specific planning sessions, and regular team review should be carried out.

### Preparation for Actual Transplant

The surgical team should comprise at least four experienced microsurgeons—two lead and two assisting surgeons who also double up for donor hand dissection. This core team should undergo repeated sessions of patient-specific planning and possibly cadaveric dissections to plan regarding the procedure, foresee any complications, and take steps to mitigate them. Cadaveric dissections and mock surgeries to mimic the registered patient, clarify the steps in everybody's minds, and ensure that everyone in the team is on the same page. It is important to take regular inventory of operating theatre (OT) instrumentation and consumables required for the transplant. Do not not forget the prosthesis for the donor, icebox to carry the hand in, ice packs, institutional permission letters to harvest hands among other things. Postoperative intensive care unit care and therapy protocol should be thought of in advance. Simultaneous public awareness programs encourage more potential patients in the OPD. In public institutions, arranging finances may be a huge challenge as the patients themselves may not be able to support the expenses involved, particularly related to the immunosuppressant drugs, outstation travel for retrieval, specific investigations like human leukocyte antigen crossmatching, serum Tacrolimus levels, etc. In these cases, the financial support from social institutions, governmental and nongovernmental charitable organizations, and crowdfunding is helpful.

The use of social networking groups on various apps (like “WhatsApp” etc.) for fast and focused coordination with the whole team is a blessing indeed. Our final aim should be capacity building and keep an optimum level of preparedness till the actual surgery happens. Formation of SOP is a major step toward ensuring training and preparedness and mock drills and dry runs ought to be done repeatedly to reinforce the SOP in the mind of every team member. Efforts should be made to complete every step that *can* be completed before the day of surgery.

The hand retrieval team plays a crucial role in the first step of the surgery. There should be adequate planning and funds should be kept ready for the retrieval teams as they may need to travel even to other cities as per the availability of the donors. In India, transplant coordinators along with Regional Organ Transplant Organization play an important part in organizing and coordinating between the harvest teams so that the whole process may proceed smoothly. The state appropriate transplant authority helps in creating a “Green Corridor” (dedicated rapid transit route cleared out for an ambulance) for the expedited transport of the harvested limb from the donor hospital to the recipient. It is helpful to be conversant with the procedure and having a dry run to make things more efficient on the day of actual surgery.

### Standard Operating Procedure

The authors believe that any complicated stream of operations may be broken into smaller steps which are easy to follow by each and everyone involved. An SOP helps to standardize the process, minimize interpersonal variations, ensures repeatability, and helps to plan in greater detail to mitigate the reasons for failure in any operation. The senior author (V.P.) has created an SOP for the department which is born out of multiple sessions of brainstorming and mock drills [Pages 1–5, SOP]. The authors believe that with minor adjustment for local conditions, the SOP can be broadly utilized by any team starting out on this journey of transplanting hands.

## Actual Transplant: What to Expect

The actual surgery is a long one. From the time the first call arrives, to the time the surgery ends, it is a roller-coaster ride. It helps to depute manpower to manage arrangement of things outside the OT and someone to log the entry and exit of the personnel since the sheer number of people involved is huge [Annexure E]. Food, rehydration, and toilet breaks can be strategically planned to prevent surgeon burnout while also not compromising on surgical results. It is a surgical endeavor which requires full commitment from the team who need to deliver with no holds barred. Postoperative coordination with therapists is essential as they are the last runners carrying the baton toward completion. It is useful to coordinate with other hand transplant units in India and abroad, to learn from their mistakes and to discuss any problems that may emerge. Finally, it helps to develop a personal rapport with patient. After this procedure, the patient becomes a part of your team and your brand ambassador, instilling confidence in other patients, showcasing what is achievable through this surgery. They become your patients for life.

## Conclusion

Starting a hand transplant program may seem to be a humongous undertaking at first but it is surely doable by breaking the process into small manageable steps. The authors hope that the SOPs attached will assist in standardizing the process and making this journey less difficult for all those who venture. The preparations start early and continue till the patient is operated.

## Summary

1. Believe in VCA
2. Preparation for transplant license
3. Form a multidisciplinary team
4. Preparation of the team
  - Read literature; Workshops; Peer groups
  - Cadaveric workshops; Mock surgeries
  - Team meetings; Repeated planning
5. Preparation of the patient
  - Counselling regarding lifelong immunosuppression
  - Investigations and references
  - Arrange funds
  - Patient registration
6. Form and follow SOP
