# Supplementary material for: Towards Starting a Hand Transplant Unit and Achieving Success in a Hand Transplant: The Standard Operating Procedure
Source: Arch Plast Surg. 2024 Feb 29;51(3):342–5. doi: 10.1055/s-0043-1776435 (PMC11081720; doi:10.1055/s-0043-1776435)
Supplement: Supplementary file 1 — Supplementary Material [file 10-1055-s-0043-1776435-s23apr0321com.pdf]

Supplementary Material S1: Checklist for preoperative evaluation of the patient

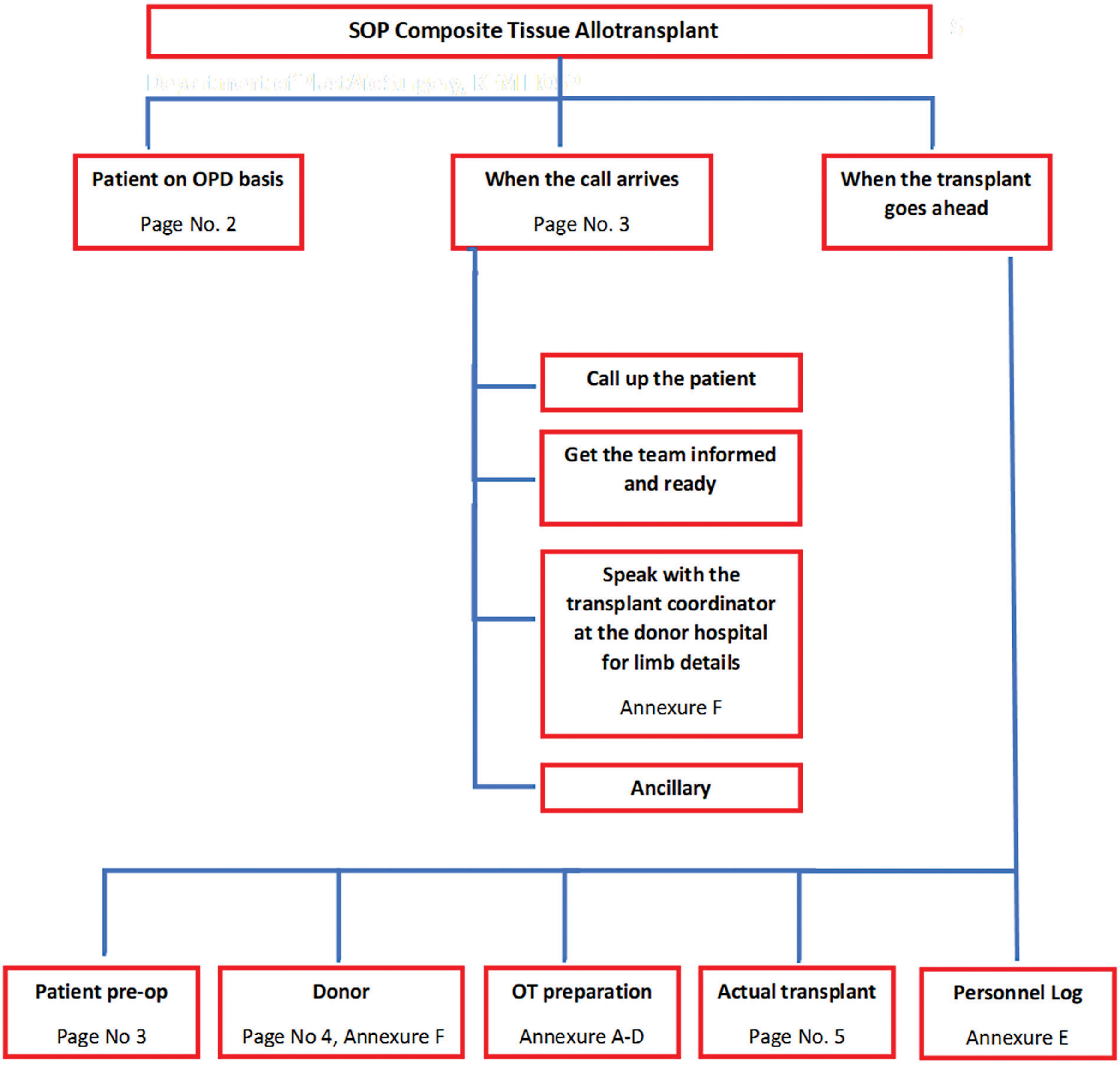

SOP for Hand Transplant

Page 1: Preoperative Evaluation in Outpatient Department

- History/Inspection/Examination
- Counselling patient with relatives
- Advise prosthesis
- Refer to therapist
- Investigations to be done
  1. For general fitness
     - (a) Complete blood count, bleeding time, clotting time
     - (b) Hematology profile
     - (c) HIV, HbSAg, HCV status
     - (d) Serum IgM, IgG
     - (e) Cytomegalovirus status
     - (f) Blood grouping
     - (g) Blood sugar level—fasting and after meal
     - (h) Renal function test
     - (i) Liver function test
     - (j) Serum sodium, potassium, chloride, calcium, and phosphorus
     - (k) Urine routine microscopy
     - (l) Urine culture

 © 2023. The Author(s).

(m) Arterial blood gas analysis
(n) X-ray chest posterior–anterior view
(o) Electrocardiography
(p) 2-D echocardiography and any other cardiac test advised by cardiologist
(q) Pulmonary function test

2. Limb specific
   (a) X-ray of upper limbs (1:1)
   (b) Arteriovenous Doppler of both upper limbs
   (c) DSA of both upper limbs
   (d) Electrodiagnostic studies of both upper limbs
   (e) Evaluation of muscle function

- References to be done
  i. Psychiatry reference
  ii. Cardiology reference
  iii. Chest medicine reference
  iv. Ophthalmology reference
  v. Skin reference
  vi. Dental evaluation
  vii. Otorhinolaryngology evaluation
  viii. Nephrology evaluation
  ix. Dermatology reference
  x. Meeting with social worker (MSW)
- Issue cost certificate and send patient to MSW for arranging money
- Photographs/Video of function of activities of daily living
- Get anesthesia and nephrology fitness done
- Carry out video consent
- Fill the form for registration and get permission from the Dean
- Register with the state organ transplant authority
- Inform ROTTO SOTTO (Regional and State Organ Transplant Organization)
- Keep copy of investigations and blood group (etc.) in the file in the department
- Repeat investigations every 6 months
- Follow-up every 3 months

Page 2: When the Call for Available Donor Arrives

(A) Call up patient
- Ask for history of fitness on phone itself
- Ask how long he/she will take to arrive
- Get him admitted
- Get investigations done
- Take preop normal and video consent again
- Ask for preop loading drugs

(B) On phone, ask MSW (transplant coordinator) in the donor hospital to check for history of contraindications for hand transplant, especially limb-related contraindications (Annexure F).
Also ask them to get X-ray of the limbs done and send to us
Limb-specific contraindications
- Any earlier injury
- Limb paralysis
- Peripheral neuropathy
- Rheumatoid arthritis or significant osteoarthritis
- Connective tissue disease
- Vasculopathy

PLUS check for Annexure F: Criteria
(C) Get the team informed and ready
- Plastic
- Anesthesia
- Ortho (for hand transplant)/ENT (for face transplant)
- Nurses
- OT support staff
- Matron
- Administration

Ancillary

Ancillary Stuff in OT and Pre-OT

Designated responsibility: Plastic Surgery Residents

1. Get all instruments checked and autoclaved
2. Check tourniquets
3. Check that implants are correct and sterilized
4. Confirm Ortho instruments
5. Check that there is 20 L ice
6. Check for Wisconsin solution
7. Arrange for refreshments as needed
8. Arrange for prescriptions of medical drugs
9. Coordinate with other departments (-Orthopaedics/Nephrology/ENT)
10. Get the ICU ready to receive the patient
11. Log in and out each person involved in the surgery: by a designated personnel (Annexure E)

Page 3: Donor Team

When team reaches the hospital for harvest

- Examine the donor for scars/evidence of marrow fracture, etc.
- Check X-ray again
- Confirm donor criteria (Annexure F)

Donor Evaluation

1. Laboratory Investigations
   - Hemoglobin and total blood count
   - Serum electrolytes
   - Blood sugar
   - Renal function tests
   - Liver function tests
   - Prothrombin time
   - Blood grouping
2. Serology
   - HIV
   - HbSAg
   - Hepatitis C
3. Tissue Typing
   - Human leukocyte antigen

 © 2023. The Author(s).

4. Imaging/Miscellaneous Tests
   - Electrocardiogram
   - Ultrasound abdomen
   - Echocardiogram
   - Chest X-ray
   - Angiography

Page 4: In OT

- Prepare two theaters
- Instruments required—Plastic Surgery (Annexure A) and Ortho (Annexure B)

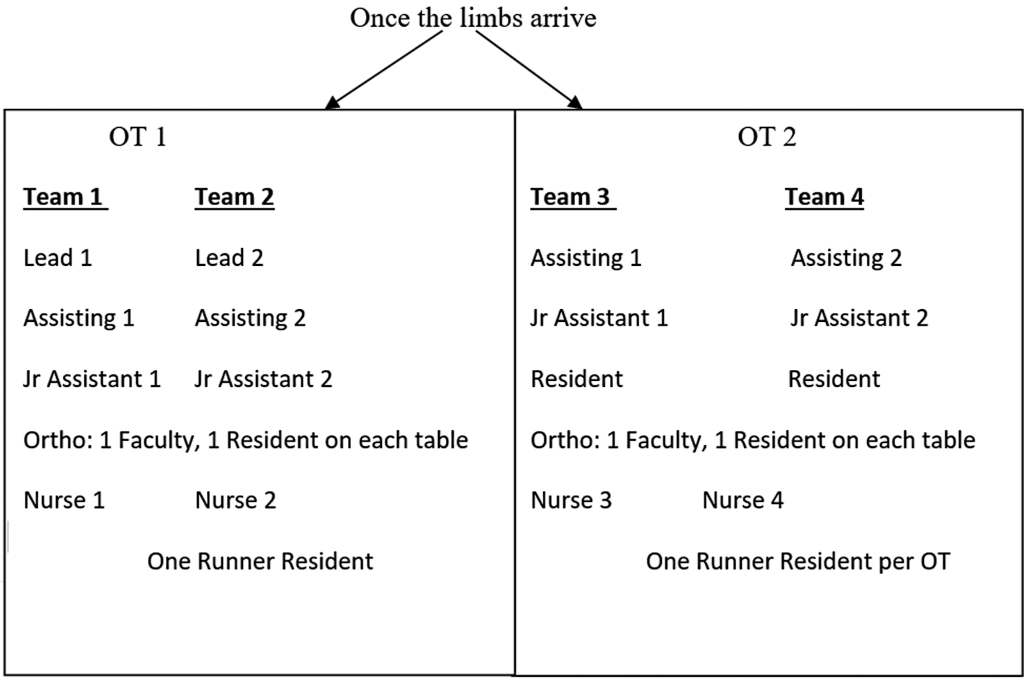

Patient induced and dissection begins

Limbs: dissect all structures and label

- One common runner resident outside OT

Actual transplant in OT 1

Team 1 + **Team 4**—Lead 1/Assisting 1/Resident 1

Senior resident to rest and come back to relieve surgeons as needed

Team 2 + **Team 3**—Lead 2/Assisting 2/Resident 2

- Immunosuppressants required: Annexure C
- Anesthesia drugs required: Annexure D

 © 2023. The Author(s).

Annexures A and B: Instruments

Sr. No.	Instruments
	General Instruments
1	Blade 15 No
2	Blade 23 No
3	B. P. Handle 3 No.
4	B. P. Handle 3 No.
5	Artery Forceps 6 Inch
6	Mosquito Forceps 3 Inch
7	Mosquito Forceps 4 Inch
8	Dissecting Forceps Plain Adson's
9	Dissecting Forceps Tooth Adson's
10	Normal Tooth Forceps
11	Normal Plain Forceps
12	Stout Scissors
13	Stille's Scissors
14	Dolphin Scissors
15	Right-Handed Retractor 1 inch
16	Right-Angled Retractor 1.5 inch
17	Right-Angled Retractor 2 inch
18	Needle Holder 3–0
19	Needle Holder 4–0
20	Needle Holder 5–0
21	Small Bone Nibbler
22	Periosteum Elevator
23	Cats Paw Retractor
24	Skin Hook
25	Sponge Holder
26	Meixter
27	Gigli Saw Handle
28	Gigli Saw
29	Allis Forceps
30	Bone Chisel
31	Wire Cutter
32	Retractor
33	Hammer
1	Scissor
2	Forceps
3	Needle Holder
4	Vessel dilator
5	Single Vascular Clamp Small
6	Single Vascular Clamp Medium

Annexure B: Orthopaedic instruments

Donor ×2 sets

1. Tourniquet
2. Periosteum elevator
3. Spike and Retractors
4. Saw with small blades (Bell)
5. Osteotomes
6. Hammer
7. Hand with drill bit
8. K-wires
9. Gigli saw (four wires)

Recipient Theater

A. Donor limb **B. Recipient limb**

1. S.S. scale (ruler)
2. Periosteum Elevator
3. Spikes and Retractors
4. Saw with small blades
5. Osteotome: 5 and 10 mm
6. Hammer
7. Hand drill with drill bits
8. Long locking 3.5 mm T plates + screws
9. LC.-DCP. Locking plates + Screws
10. 3.5 mm (small fragment) fixation set

 © 2023. The Author(s).

Annexure C: Immunosuppressants Required

(A) List of drugs

1. Injection, Thymoglobulin 1.5 mg/kg per dose
2. Injection, Methylprednisolone 1000, 500, and 250 mg
3. Injection, Human albumin 20%
4. Injection, Papaverine
5. Capsule, Tacrolimus 1 mg
6. Capsule, Tacrolimus 0.5 mg
7. Tablet, Mycophenolate 50 mg
8. Tablet, Prednisolone 5, 10, 20, and 30 mg
9. Tablet, Valganciclovir 450 mg
10. Tablet, Cotrimoxazole
11. Tablet, Diclofenac
12. Benzoic acid powder

(B) Immunosuppression regimen

- Induction regime

Day (−1)

- Injection, Thymoglobulin 1.5 mg/kg
- Injection, Methylprednisolone 500 mg stat
- Capsule, Tacrolimus 0.05 mg/kg stat
- Tablet, Mycophenolate mofetil 1000 mg stat
- Tablet, Valganciclovir 900 mg OD
- Injection, Piperacillin Tazobactam 4.5 g IV stat
- Before release of vascular clamp
- Injection, Methylprednisolone 500 mg stat

Day (0)

- Injection Thymoglobulin 1.5 mg/kg
- Injection Methylprednisolone 250 mg stat
- Capsule, Tacrolimus 0.1 mg/kg in two divided doses (8 AM and 8 PM)
- Tablet, Mycophenolate mofetil 1,000 mg twice daily (8 AM and 8 PM)
- Tablet, Valganciclovir 450 mg OD
- Injection, Piperacillin Tazobactam 4.5 g IV (three times a day)
- Candid mouth paint (four times a day)

Day 1 onwards

- Injection, Thymoglobulin 1.5 mg/kg for 3 days
- Tablet, Prednisolone 0.5 mg/kg/day
- Capsule, Tacrolimus 0.1 mg/kg in two divided doses (8 a.m. and 8 p.m.)
- Tablet, Mycophenolate mofetil 1,000 mg twice daily (8 a.m. and 8 p.m.)
- Tablet, Valganciclovir 450 mg OD
- Injection, Piperacillin Tazobactam 4.5 g IV, three times a day, for 5 days
- Candid mouth paint (four times a day)

Maintenance regime

- Tablet, Prednisolone 0.5 mg/kg/day
- Capsule, Tacrolimus 0.1 mg/kg in two divided doses (8 a.m. and 8 p.m.)
- Tablet, Mycophenolate mofetil 1,000 mg twice daily (8 a.m. and 8 p.m.)
- Tablet, Valganciclovir 450 mg OD
- Tablet, Fluconazole 100 mg OD from second week onwards
- Tablet, Nifedipine 20 mg (three times a day) from sixth week onwards

 © 2023. The Author(s).

Annexure D: Anesthesia consumables

SR NO	Item	Quantity
1		
	16G	6
	18G	6
	20G	6
	22G	6
2		
	Micro drip	6
	Macro drip	6
	Blood transfusion set	6
3	IV infusion fluid	
	NS	8
	RL	8
	D5	5
	DNS	5
	Tetrastarch	5
	Dextran	2
	Blood	5
	Platelets	5
	FFP	5
4	Antibiotics	4
5	ECG leads	20
6		
	3 inch	1
	1½ inch	1
	1 inch	1
7	Dynaplast	1
8	Transpore 1 inch	1
9	Durapore 1 inch	1
10	Tegaderm 8*16 cm	10
11	Antibiotic-impregnated dressings	5
12	Sterile polydrapes	
	Small	10
	Medium	10
	Large	10
13	Three-way bivalve	10
14	Venous extension	
	10 cm	10
	50 cm	10
	100 cm	10
	200 cm	10
15	Arterial Ext. monitoring line	
	100 cm	10
	200 cm	10
16	Stimuplex Needle	
	5 cm	5
	10 cm	5
17	Peripheral Nerve Locator	1
18	USG machine	
19	USG jelly	
20	Contiplex Catheter	
20	Drugs	
	Local anesthetics	
	Lignocaine 2% plain	5
	Lignocaine 2%	5
	Lignocaine 2% preservative free	2
	Bupivacaine 0.25%	5
	Bupivacaine 0.5%	5
	Ropivacaine 0.2%	5
	Ropivacaine 0.5%	5
	Ropivacaine 0.75%	5
21	Additives	
	Clonidine	5
	Buprenorphine	5
	Dexamethasone	5
	Soda-bicarb	5
22	Relaxants	
	Vecuronium/Atracurium/ Rocuronium	10 each
23	Opioids	
	Fentanyl	10
24	Dexmedetomidine	5
	Propofol 50 mL	5
	Propofol 20 mL	5
	Thiopentone	5
	Ketamine	5
	Etomidate	5
26	Sedatives	
	Midazolam	10
27	Ondansetron	5
28	Inhalational agents	
	Isoflurane	3
	Sevoflurane	3
	Desflurane	3
29	Heparin	5
	Protamine	5
30	Paracetamol	5
	Tramadol	5
	Diclofenac	5
31	Asthaline pump	2
32	Duoline respules	5
33	Budecort respules	5
34	Hotline warming set	5
35	Fluid warmers	1
36	Warming mattress	2
37	Pressure bags	5
39	Infusion pumps	5
40	Elastomeric pump	5
41	Ryles tube	
	12F	5
	14F	5
	16F	5

 © 2023. The Author(s).

Annexure E: Personnel Log

Logged by

Name of Logger	Signature

Name of personnel	Department	In-time	Out-time	Break

Annexure F: Criteria

Limb-specific contraindications

- Limb paralysis
- Peripheral neuropathy
- Rheumatoid arthritis or significant osteoarthritis
- Connective tissue disease
- Vasculopathy

Donor Selection/Inclusion Criteria

i. Brain death certification
ii. Hemodynamically stable with optimal ionotropic support
iii. Age < 55 years
iv. No significant coronary artery disease (coronary angiography indicated for age > 40 years)
v. No significant structural heart disease (Echocardiogram required)
vi. Ejection fraction, more than 45% by Echo
vii. No sepsis
viii. No malignancy
ix. ABO compatibility
x. Weight/size matching
xi. Appropriate consent for organ donation

Donor Exclusion Criteria

i. Significant cardiac anomalies
ii. History of myocardial infarction (MI)
iii. Significant valvular abnormalities
iv. Refractory ventricular arrhythmia
v. Ejection fraction, less than 45% by Echo.
vi. Bacterial/fungal sepsis
vii. Seropositive status—HIV, HBsAg, Hepatitis C, etc., positive
viii. History of drug abuse
ix. ABO incompatibility
x. Size/Weight mismatch
xi. Hand-specific

Inclusion criteria	Exclusion criteria
Donor age, similar to recipient	Absolute
Donor sex, same as recipient	Risk of infection transmission
Donor blood type, compatible with recipient	HIV
Donor skin color and hair pattern, similar to recipient	Hepatitis B/C
No identifying marks on donor limb	Current malignancy
Same EBV and CMV status of donor and recipient limb	Limb paralysis
	Peripheral neuropathy
	RA or significant osteoarthritis
	Connective tissue disease
	**Relative**
	Viral encephalitis
	Uncontrolled HTN

Supplementary Material S2: SOP and Annexures for the Hand Transplant Team

Hand transplant checklist

Name:
Age/Sex:
Address:
Contact:
Email:
Trauma etiology:
Date of trauma:
Laterality:
Level of amputation:
Prosthesis use:
Blood group:

Preoperative Evaluation in Outpatient Department

- History/Inspection/Examination
- Counselling patient with relatives
- Consent: Photographs/Video of function of activities of daily living or Carry out video consent
- Advice prosthesis or Refer to therapist

 © 2023. The Author(s).

- **Investigations**

(A) For general fitness

- ➢ Complete blood count, bleeding time, clotting time
- ➢ Hematology profile
- ➢ HIV, HbsAg, HCV status
- ➢ Blood grouping
- ➢ Blood sugar—fasting and after meal
- ➢ Renal function test
- ➢ Liver function test
- ➢ Serum sodium, potassium, chloride, calcium, and phosphorus
- ➢ Urine routine microscopy
- ➢ Urine culture
- ➢ Arterial blood gas analysis
- ➢ X-ray chest posterior–anterior view
- ➢ Electrocardiography
- ➢ 2-D echocardiography and any other cardiac test advised by cardiologist
- ➢ Pulmonary function test
- ➢ Panel reactive antibody
- ➢ Hep B vaccine: 0, 1, and 6 months
- ➢ Influenza vaccine: 0 and 1 year
- ➢ Pneumococcal vaccine: 0 and 2 months
- ➢ Covid vaccination: single, double, booster dose
- ➢ USG abdomen/pelvis

(B) Limb specific

- ➢ X-ray of upper limbs (1:1)
- ➢ Arteriovenous Doppler of both upper limbs
- ➢ DSA of both upper limbs
- ➢ Electromyography and nerve conduction studies (EMG/NCS) of both upper limbs (if needed)
- ➢ MRI of amputated stump to look for nerves and muscle
- ➢ Evaluation of muscle function via charting

- References for fitness for SURGERY and lifelong IMMUNOSUPPRESSION

- ➢ Psychiatry reference for mental stability regarding high risk and lengthy procedure which has life-altering implications
- ➢ Cardiology reference
- ➢ Chest medicine reference
- ➢ Ophthalmology reference
- ➢ Skin reference
- ➢ Dental evaluation
- ➢ ENT evaluation
- ➢ Nephrology evaluation—cross-check about all investigations needed
- ➢ Dermatology reference
- ➢ Orthopaedic reference
- ➢ OB/GYN reference for female patients
- ➢ GI medicine reference
- ➢ GI surgery reference
- ➢ Meeting with social worker
- ➢ Get anesthesia fitness done

- Issue cost certificate and send patient to MSW for arranging money and from CM fund of the home state he is domiciled in.
- Registration
  - ➢ Fill the form for registration and get permission from the Dean
  - ➢ Register with appropriate authority
  - ➢ Keep copy of investigations and blood group, etc., in the file in our department
  - ➢ Repeat investigations every 6 months
  - ➢ Follow-up every 3 months

 © 2023. The Author(s).
